# Supplementary material for: Modeling glioblastoma heterogeneity as a dynamic network of cell states
Source: Mol Syst Biol. 2021 Sep 16;17(9):e10105. doi: 10.15252/msb.202010105 (PMC8444284; doi:10.15252/msb.202010105)
Supplement: Supplementary file 5 — Source Data for Figure 3 [file MSB-17-e10105-s001.zip › Figure3A_sourcedata/GSEA_3065/nosigdb_state4.GseaPreranked.1622718430994/RICHARDS_ZHONG_NATURE_2018_ASTROCYTES_UPREG.html]

Details for gene set RICHARDS\_ZHONG\_NATURE\_2018\_ASTROCYTES\_UPREG[GSEA]

|  || Dataset | state4 |
| Phenotype | NoPhenotypeAvailable |
| Upregulated in class | na\_pos |
| GeneSet | RICHARDS\_ZHONG\_NATURE\_2018\_ASTROCYTES\_UPREG |
| Enrichment Score (ES) | 0.4427579 |
| Normalized Enrichment Score (NES) | 2.0807316 |
| Nominal p-value | 0.0 |
| FDR q-value | 1.5454544E-4 |
| FWER p-Value | 0.001 |
Table: GSEA Results Summary

  

Fig 1: Enrichment plot: RICHARDS\_ZHONG\_NATURE\_2018\_ASTROCYTES\_UPREG      
 Profile of the Running ES Score & Positions of GeneSet Members on the Rank Ordered List

  

| PROBE | GENE SYMBOL | GENE\_TITLE | RANK IN GENE LIST | RANK METRIC SCORE | RUNNING ES | CORE ENRICHMENT || 1 | GFAP |  |  | 0 | 0.995 | 0.0370 | Yes |
| 2 | FABP7 |  |  | 5 | 0.656 | 0.0608 | Yes |
| 3 | S100B |  |  | 6 | 0.630 | 0.0842 | Yes |
| 4 | SPARCL1 |  |  | 17 | 0.416 | 0.0980 | Yes |
| 5 | OLFM2 |  |  | 20 | 0.396 | 0.1124 | Yes |
| 6 | RAB31 |  |  | 21 | 0.388 | 0.1269 | Yes |
| 7 | FABP5 |  |  | 23 | 0.375 | 0.1406 | Yes |
| 8 | HOPX |  |  | 28 | 0.360 | 0.1534 | Yes |
| 9 | QKI |  |  | 31 | 0.345 | 0.1659 | Yes |
| 10 | MT3 |  |  | 38 | 0.318 | 0.1767 | Yes |
| 11 | HEY1 |  |  | 39 | 0.318 | 0.1885 | Yes |
| 12 | SOX9 |  |  | 48 | 0.299 | 0.1983 | Yes |
| 13 | TSC22D4 |  |  | 49 | 0.298 | 0.2094 | Yes |
| 14 | SNX3 |  |  | 51 | 0.297 | 0.2202 | Yes |
| 15 | C1orf122 |  |  | 53 | 0.294 | 0.2310 | Yes |
| 16 | ID2 |  |  | 56 | 0.292 | 0.2416 | Yes |
| 17 | DKK3 |  |  | 66 | 0.281 | 0.2505 | Yes |
| 18 | PLEKHB1 |  |  | 82 | 0.262 | 0.2578 | Yes |
| 19 | ID1 |  |  | 84 | 0.261 | 0.2673 | Yes |
| 20 | LGALS3 |  |  | 89 | 0.259 | 0.2763 | Yes |
| 21 | CLU |  |  | 95 | 0.252 | 0.2848 | Yes |
| 22 | TTYH1 |  |  | 100 | 0.249 | 0.2934 | Yes |
| 23 | PEA15 |  |  | 112 | 0.243 | 0.3007 | Yes |
| 24 | FAM181B |  |  | 130 | 0.232 | 0.3065 | Yes |
| 25 | ZFP36L2 |  |  | 135 | 0.228 | 0.3143 | Yes |
| 26 | ENHO |  |  | 136 | 0.228 | 0.3228 | Yes |
| 27 | GATM |  |  | 156 | 0.219 | 0.3278 | Yes |
| 28 | ATP1B2 |  |  | 159 | 0.217 | 0.3355 | Yes |
| 29 | CNN3 |  |  | 160 | 0.217 | 0.3436 | Yes |
| 30 | RHOC |  |  | 169 | 0.214 | 0.3502 | Yes |
| 31 | TRIM47 |  |  | 175 | 0.212 | 0.3573 | Yes |
| 32 | HSPB1 |  |  | 180 | 0.211 | 0.3645 | Yes |
| 33 | LRRN3 |  |  | 181 | 0.211 | 0.3723 | Yes |
| 34 | CBR1 |  |  | 192 | 0.206 | 0.3783 | Yes |
| 35 | ITPR2 |  |  | 222 | 0.193 | 0.3807 | Yes |
| 36 | GNG12 |  |  | 231 | 0.190 | 0.3865 | Yes |
| 37 | EDNRB |  |  | 271 | 0.180 | 0.3868 | Yes |
| 38 | UBL3 |  |  | 278 | 0.179 | 0.3924 | Yes |
| 39 | LITAF |  |  | 295 | 0.174 | 0.3963 | Yes |
| 40 | KLHDC8A |  |  | 300 | 0.174 | 0.4021 | Yes |
| 41 | GPM6B |  |  | 346 | 0.166 | 0.4008 | Yes |
| 42 | TP53I3 |  |  | 353 | 0.164 | 0.4059 | Yes |
| 43 | ABAT |  |  | 435 | 0.145 | 0.3980 | Yes |
| 44 | SEMA6D |  |  | 454 | 0.143 | 0.4003 | Yes |
| 45 | GDPD2 |  |  | 477 | 0.141 | 0.4019 | Yes |
| 46 | ZFP36L1 |  |  | 497 | 0.137 | 0.4039 | Yes |
| 47 | HADHB |  |  | 503 | 0.136 | 0.4081 | Yes |
| 48 | LAMB2 |  |  | 510 | 0.135 | 0.4121 | Yes |
| 49 | IFITM3 |  |  | 546 | 0.132 | 0.4112 | Yes |
| 50 | APOE |  |  | 569 | 0.129 | 0.4124 | Yes |
| 51 | MLC1 |  |  | 594 | 0.126 | 0.4131 | Yes |
| 52 | CDC42EP4 |  |  | 600 | 0.125 | 0.4169 | Yes |
| 53 | WSCD1 |  |  | 605 | 0.125 | 0.4209 | Yes |
| 54 | PLTP |  |  | 625 | 0.123 | 0.4223 | Yes |
| 55 | MYO10 |  |  | 673 | 0.118 | 0.4190 | Yes |
| 56 | BDH2 |  |  | 688 | 0.116 | 0.4210 | Yes |
| 57 | GNG7 |  |  | 718 | 0.112 | 0.4203 | Yes |
| 58 | NTRK2 |  |  | 722 | 0.111 | 0.4240 | Yes |
| 59 | EFEMP2 |  |  | 727 | 0.111 | 0.4274 | Yes |
| 60 | PMP22 |  |  | 765 | 0.107 | 0.4253 | Yes |
| 61 | CST3 |  |  | 792 | 0.104 | 0.4248 | Yes |
| 62 | C1orf61 |  |  | 803 | 0.104 | 0.4270 | Yes |
| 63 | ANXA5 |  |  | 814 | 0.103 | 0.4292 | Yes |
| 64 | GLUL |  |  | 819 | 0.102 | 0.4324 | Yes |
| 65 | SHISA4 |  |  | 848 | 0.099 | 0.4314 | Yes |
| 66 | GNG11 |  |  | 857 | 0.099 | 0.4338 | Yes |
| 67 | ZFYVE21 |  |  | 956 | 0.091 | 0.4210 | Yes |
| 68 | SOX2 |  |  | 975 | 0.090 | 0.4213 | Yes |
| 69 | RGMA |  |  | 995 | 0.089 | 0.4215 | Yes |
| 70 | HTRA1 |  |  | 1008 | 0.088 | 0.4228 | Yes |
| 71 | CSPG5 |  |  | 1035 | 0.086 | 0.4217 | Yes |
| 72 | DOK5 |  |  | 1052 | 0.084 | 0.4222 | Yes |
| 73 | NOTCH3 |  |  | 1061 | 0.084 | 0.4239 | Yes |
| 74 | DTNA |  |  | 1063 | 0.084 | 0.4269 | Yes |
| 75 | SLC1A3 |  |  | 1069 | 0.083 | 0.4292 | Yes |
| 76 | SEMA6A |  |  | 1073 | 0.083 | 0.4318 | Yes |
| 77 | EMC2 |  |  | 1076 | 0.083 | 0.4345 | Yes |
| 78 | RASSF4 |  |  | 1099 | 0.082 | 0.4339 | Yes |
| 79 | AGT |  |  | 1103 | 0.082 | 0.4365 | Yes |
| 80 | ADD3 |  |  | 1118 | 0.081 | 0.4372 | Yes |
| 81 | AIF1L |  |  | 1119 | 0.081 | 0.4402 | Yes |
| 82 | DDAH1 |  |  | 1153 | 0.078 | 0.4377 | Yes |
| 83 | SLC4A4 |  |  | 1160 | 0.078 | 0.4396 | Yes |
| 84 | HES4 |  |  | 1197 | 0.076 | 0.4365 | Yes |
| 85 | ETV1 |  |  | 1199 | 0.076 | 0.4391 | Yes |
| 86 | CA12 |  |  | 1205 | 0.076 | 0.4411 | Yes |
| 87 | TFPI |  |  | 1213 | 0.075 | 0.4428 | Yes |
| 88 | RAMP1 |  |  | 1283 | 0.072 | 0.4340 | No |
| 89 | GLUD1 |  |  | 1295 | 0.071 | 0.4348 | No |
| 90 | GNPTG |  |  | 1299 | 0.071 | 0.4370 | No |
| 91 | RAB34 |  |  | 1330 | 0.069 | 0.4346 | No |
| 92 | VIM |  |  | 1350 | 0.069 | 0.4340 | No |
| 93 | APBB2 |  |  | 1358 | 0.068 | 0.4354 | No |
| 94 | SLC25A18 |  |  | 1363 | 0.068 | 0.4373 | No |
| 95 | FERMT2 |  |  | 1426 | 0.065 | 0.4294 | No |
| 96 | SPRY1 |  |  | 1437 | 0.065 | 0.4302 | No |
| 97 | HES1 |  |  | 1442 | 0.065 | 0.4319 | No |
| 98 | SERPINB6 |  |  | 1472 | 0.063 | 0.4295 | No |
| 99 | TRIB2 |  |  | 1496 | 0.062 | 0.4280 | No |
| 100 | LRP4 |  |  | 1502 | 0.062 | 0.4295 | No |
| 101 | PBXIP1 |  |  | 1539 | 0.060 | 0.4258 | No |
| 102 | MT2A |  |  | 1542 | 0.060 | 0.4277 | No |
| 103 | TSPAN3 |  |  | 1584 | 0.059 | 0.4231 | No |
| 104 | BBS2 |  |  | 1643 | 0.057 | 0.4156 | No |
| 105 | MRC2 |  |  | 1647 | 0.057 | 0.4173 | No |
| 106 | PRCP |  |  | 1675 | 0.056 | 0.4149 | No |
| 107 | TM7SF2 |  |  | 1689 | 0.056 | 0.4148 | No |
| 108 | GNG5 |  |  | 1709 | 0.055 | 0.4137 | No |
| 109 | SEZ6L |  |  | 1768 | 0.053 | 0.4061 | No |
| 110 | ALDH6A1 |  |  | 1803 | 0.052 | 0.4024 | No |
| 111 | SASH1 |  |  | 1848 | 0.051 | 0.3970 | No |
| 112 | C2orf72 |  |  | 1897 | 0.049 | 0.3909 | No |
| 113 | CRYL1 |  |  | 1922 | 0.049 | 0.3888 | No |
| 114 | DDR1 |  |  | 1941 | 0.048 | 0.3876 | No |
| 115 | LIFR |  |  | 1972 | 0.048 | 0.3844 | No |
| 116 | PLIN3 |  |  | 1974 | 0.048 | 0.3860 | No |
| 117 | GRB14 |  |  | 2026 | 0.046 | 0.3793 | No |
| 118 | PDLIM3 |  |  | 2070 | 0.045 | 0.3739 | No |
| 119 | OAT |  |  | 2106 | 0.044 | 0.3697 | No |
| 120 | PREX1 |  |  | 2110 | 0.044 | 0.3709 | No |
| 121 | TECPR2 |  |  | 2115 | 0.043 | 0.3718 | No |
| 122 | PSAT1 |  |  | 2153 | 0.043 | 0.3673 | No |
| 123 | NLGN3 |  |  | 2183 | 0.042 | 0.3640 | No |
| 124 | SDC3 |  |  | 2214 | 0.041 | 0.3606 | No |
| 125 | PHYHIPL |  |  | 2328 | 0.038 | 0.3434 | No |
| 126 | SLC38A3 |  |  | 2359 | 0.038 | 0.3398 | No |
| 127 | SPRY2 |  |  | 2432 | 0.036 | 0.3293 | No |
| 128 | SNX5 |  |  | 2437 | 0.036 | 0.3300 | No |
| 129 | TSPAN6 |  |  | 2443 | 0.036 | 0.3305 | No |
| 130 | RFX4 |  |  | 2454 | 0.036 | 0.3302 | No |
| 131 | PPP1R16A |  |  | 2513 | 0.034 | 0.3219 | No |
| 132 | PDLIM5 |  |  | 2517 | 0.034 | 0.3226 | No |
| 133 | GPRC5B |  |  | 2596 | 0.033 | 0.3110 | No |
| 134 | CAMK2G |  |  | 2599 | 0.033 | 0.3119 | No |
| 135 | LIPA |  |  | 2649 | 0.032 | 0.3049 | No |
| 136 | HMGCS1 |  |  | 2674 | 0.031 | 0.3021 | No |
| 137 | BMPR1B |  |  | 2711 | 0.030 | 0.2973 | No |
| 138 | YIF1A |  |  | 2723 | 0.030 | 0.2966 | No |
| 139 | IDI1 |  |  | 2749 | 0.030 | 0.2936 | No |
| 140 | GPR137B |  |  | 2769 | 0.029 | 0.2915 | No |
| 141 | ABCD3 |  |  | 2800 | 0.029 | 0.2876 | No |
| 142 | SIRPA |  |  | 3078 | 0.023 | 0.2427 | No |
| 143 | LRIG1 |  |  | 3101 | 0.023 | 0.2400 | No |
| 144 | PHACTR2 |  |  | 3125 | 0.023 | 0.2370 | No |
| 145 | F3 |  |  | 3133 | 0.022 | 0.2367 | No |
| 146 | G6PC3 |  |  | 3156 | 0.022 | 0.2339 | No |
| 147 | ROBO3 |  |  | 3248 | 0.020 | 0.2196 | No |
| 148 | S100A13 |  |  | 3355 | 0.017 | 0.2027 | No |
| 149 | LGALS1 |  |  | 3527 | 0.011 | 0.1748 | No |
| 150 | ITM2C |  |  | 3587 | 0.009 | 0.1654 | No |
| 151 | ARHGEF26 |  |  | 3596 | 0.009 | 0.1644 | No |
| 152 | TMEM132B |  |  | 3600 | 0.009 | 0.1643 | No |
| 153 | COMT |  |  | 3621 | 0.008 | 0.1612 | No |
| 154 | LEPROT |  |  | 3646 | 0.007 | 0.1576 | No |
| 155 | TNFRSF19 |  |  | 3725 | 0.004 | 0.1448 | No |
| 156 | LGALS3BP |  |  | 3757 | 0.003 | 0.1398 | No |
| 157 | EFHD2 |  |  | 3841 | -0.001 | 0.1261 | No |
| 158 | SOX8 |  |  | 3882 | -0.003 | 0.1196 | No |
| 159 | DARS |  |  | 3886 | -0.003 | 0.1192 | No |
| 160 | NOTCH2 |  |  | 3944 | -0.006 | 0.1101 | No |
| 161 | ADGRG1 |  |  | 3961 | -0.007 | 0.1077 | No |
| 162 | JUN |  |  | 3979 | -0.008 | 0.1052 | No |
| 163 | SPATA6 |  |  | 4039 | -0.012 | 0.0959 | No |
| 164 | P3H4 |  |  | 4091 | -0.015 | 0.0880 | No |
| 165 | DBI |  |  | 4122 | -0.017 | 0.0836 | No |
| 166 | GJA1 |  |  | 4123 | -0.017 | 0.0843 | No |
| 167 | CTSL |  |  | 4153 | -0.019 | 0.0802 | No |
| 168 | DCXR |  |  | 4188 | -0.021 | 0.0754 | No |
| 169 | MEIS1 |  |  | 4218 | -0.023 | 0.0714 | No |
| 170 | PDPN |  |  | 4220 | -0.023 | 0.0721 | No |
| 171 | DAG1 |  |  | 4317 | -0.030 | 0.0574 | No |
| 172 | ID4 |  |  | 4329 | -0.031 | 0.0567 | No |
| 173 | TEX264 |  |  | 4363 | -0.033 | 0.0525 | No |
| 174 | PLEC |  |  | 4408 | -0.036 | 0.0466 | No |
| 175 | MAGT1 |  |  | 4497 | -0.044 | 0.0337 | No |
| 176 | ALCAM |  |  | 4499 | -0.045 | 0.0352 | No |
| 177 | ADORA2B |  |  | 4561 | -0.049 | 0.0269 | No |
| 178 | CCDC80 |  |  | 4657 | -0.056 | 0.0133 | No |
| 179 | BCHE |  |  | 4672 | -0.057 | 0.0131 | No |
| 180 | SCD |  |  | 4717 | -0.061 | 0.0081 | No |
| 181 | BMP7 |  |  | 4727 | -0.062 | 0.0089 | No |
| 182 | P4HA1 |  |  | 4761 | -0.065 | 0.0059 | No |
| 183 | S100A16 |  |  | 4787 | -0.067 | 0.0042 | No |
| 184 | PTTG1IP |  |  | 4943 | -0.079 | -0.0185 | No |
| 185 | SCRG1 |  |  | 4970 | -0.080 | -0.0198 | No |
| 186 | HSD17B12 |  |  | 5056 | -0.087 | -0.0306 | No |
| 187 | AASS |  |  | 5154 | -0.097 | -0.0430 | No |
| 188 | CYP51A1 |  |  | 5190 | -0.101 | -0.0451 | No |
| 189 | ELOVL5 |  |  | 5226 | -0.103 | -0.0470 | No |
| 190 | PON2 |  |  | 5246 | -0.105 | -0.0462 | No |
| 191 | PTN |  |  | 5261 | -0.107 | -0.0446 | No |
| 192 | ITGAV |  |  | 5309 | -0.110 | -0.0482 | No |
| 193 | TNC |  |  | 5359 | -0.114 | -0.0521 | No |
| 194 | SFXN5 |  |  | 5408 | -0.120 | -0.0556 | No |
| 195 | ABHD3 |  |  | 5523 | -0.134 | -0.0694 | No |
| 196 | FADS2 |  |  | 5544 | -0.135 | -0.0677 | No |
| 197 | SYPL1 |  |  | 5607 | -0.145 | -0.0725 | No |
| 198 | CD82 |  |  | 5621 | -0.147 | -0.0692 | No |
| 199 | MFGE8 |  |  | 5668 | -0.154 | -0.0711 | No |
| 200 | PTPRZ1 |  |  | 5811 | -0.186 | -0.0876 | No |
| 201 | B2M |  |  | 5823 | -0.188 | -0.0824 | No |
| 202 | MSMO1 |  |  | 5866 | -0.198 | -0.0820 | No |
| 203 | FAT1 |  |  | 5898 | -0.206 | -0.0795 | No |
| 204 | SERPINE2 |  |  | 5913 | -0.210 | -0.0740 | No |
| 205 | RCN1 |  |  | 5945 | -0.219 | -0.0710 | No |
| 206 | BCAP29 |  |  | 6036 | -0.259 | -0.0762 | No |
| 207 | NT5E |  |  | 6064 | -0.277 | -0.0704 | No |
| 208 | DHCR7 |  |  | 6083 | -0.287 | -0.0627 | No |
| 209 | PHGDH |  |  | 6126 | -0.318 | -0.0578 | No |
| 210 | SLC3A2 |  |  | 6174 | -0.376 | -0.0516 | No |
| 211 | CD9 |  |  | 6176 | -0.378 | -0.0377 | No |
| 212 | TIMP1 |  |  | 6191 | -0.409 | -0.0248 | No |
| 213 | BCAN |  |  | 6211 | -0.481 | -0.0100 | No |
| 214 | CD63 |  |  | 6220 | -0.496 | 0.0071 | No |
Table: GSEA details [plain text format]

  

Fig 2: RICHARDS\_ZHONG\_NATURE\_2018\_ASTROCYTES\_UPREG: Random ES distribution      
 Gene set null distribution of ES for **RICHARDS\_ZHONG\_NATURE\_2018\_ASTROCYTES\_UPREG**
